# Indexing *TNF-α *gene expression using a gene-targeted reporter cell line

**DOI:** 10.1186/1741-7007-7-8

**Published:** 2009-02-16

**Authors:** Ziying Yan, Diana Lei-Butters, John F Engelhardt, Gregory H Leno

**Affiliations:** 1 Department of Anatomy and Cell Biology, Carver College of Medicine, University of Iowa, Iowa City, IA, USA; 2 Department of Internal Medicine, Carver College of Medicine, University of Iowa, Iowa City, IA, USA; 3 Center for Gene Therapy of Cystic Fibrosis and Other Genetic Diseases, Carver College of Medicine, University of Iowa, Iowa City, IA, USA

## Abstract

**Background:**

Current cell-based drug screening technologies utilize randomly integrated reporter genes to index transcriptional activity of an endogenous gene of interest. In this context, reporter expression is controlled by known genetic elements that may only partially capture gene regulation and by unknown features of chromatin specific to the integration site. As an alternative technology, we applied highly efficient gene-targeting with recombinant adeno-associated virus to precisely integrate a luciferase reporter gene into exon 1 of the HeLa cell tumor necrosis factor-alpha (*TNF-α*) gene. Drugs known to induce *TNF-α *expression were then used to compare the authenticity of gene-targeted and randomly integrated transcriptional reporters.

**Results:**

*TNF-α*-targeted reporter activity reflected endogenous *TNF-α *mRNA expression, whereas randomly integrated *TNF-α *reporter lines gave variable expression in response to transcriptional and epigenetic regulators. 5,6-Dimethylxanthenone-4-acetic acid (DMXAA), currently used in cancer clinical trials to induce *TNF-α *gene transcription, was only effective at inducing reporter expression from *TNF-α *gene-targeted cells.

**Conclusion:**

We conclude that gene-targeted reporter cell lines provide predictive indexing of gene transcription for drug discovery.

## Background

Transcriptional regulation provides an ideal target for therapeutic intervention. As such, tools for studying transcriptional modulators of disease genes will help to facilitate the development of novel therapeutics [1]. Cell lines have been used to study the expression of specific genes involved in disease development or at signal transduction checkpoints, and are currently a front-line approach for early-stage drug discovery. A number of indirect techniques are available to assess gene transcription in cells including ELISA and gene arrays or quantitative PCR for measuring the gene transcript levels. However, these methods are time consuming, resource intensive and/or do not directly assess the transcriptional activity of an endogenous promoter. Moreover, they are not amenable to high-throughput screening (HTS) for efficient detection of drug-induced changes in disease gene expression.

Cell-based gene reporter assay systems were developed as an alternative system amenable to HTS over 10 years ago, and have been widely used to study transcription and gene regulation. Specifically, linking easily detectable reporter genes – such as luciferase, β-galactosidase or green fluorescent protein – to defined gene promoters and regulatory elements has resulted in the production of numerous reporter vectors. Transient transfection of such reporter vectors into cultured cells and quantitative analysis of the reporter gene product is a fast and efficient way to study disease gene expression. Moreover, the establishment of cell lines containing random stable integrants has made possible the development of cell-based reporter assays [2], which have now been successfully scaled-up for HTS following advances in robotics and fluorescence/luminescence plate-reader technologies [3,4]. Recently, a novel reporter system was developed in which Flp recombinase is used to produce flippase recognition target (FRT) single site-specific integration of a reporter gene construct at a transcriptionally-active genomic locus in cultured cells [5]. This approach has several advantages over randomly integrated reporter constructs including single copy construct integration and a single chromatin context within which the effects of promoter mutations or single nucleotide polymorphisms (SNPs) on gene expression can be studied [5]. Moreover, this reporter system has been used to screen small molecules for inhibition of the pro-inflammatory cytokine, tumor necrosis factor (TNF) [6]. Although randomly integrated and FRT single site-specific reporters are presumed to reflect endogenous regulation of the disease gene, this is a questionable assumption given the unknown epigenetic influences of chromatin structure on gene transcription along with missing genetic elements that regulate gene expression at the endogenous locus. To this end, optimal systems would utilize gene-targeted reporters controlled by endogenous regulatory sequences and governed by an inherited epigenetic program unique to a given disease gene locus. Although gene targeting in mouse embryonic stem cells makes it possible to precisely integrate exogenous DNA sequence into a predetermined 'target' gene locus [7], such systems have been much less effective in somatic cells. An alternative approach, utilizing single-stranded recombinant adeno-associated virus (rAAV) to promote homologous recombination between the targeting construct and the chromosome [8-11] has been widely applied to genetically modify endogenous genes by insertion, deletion/replacement, and point mutation [11-14]. The efficiency of gene targeting using single-stranded rAAV vectors is also much higher than that observed with adenovirus- or retrovirus-based vector systems [13]. Self-complementary rAAV (scAAV) vectors have been shown to promote more efficient viral transduction than single-stranded rAAV vectors both *in vitro *and *in vivo *[15]. However, these double-stranded vectors do not appear to contribute to the gene targeting reaction [13,16].

The *TNF-α *gene maps to chromosome 6p21.3, contains four exons, and spans approximately 3 kb of DNA in human cells [17,18]. *TNF-α *gene expression is cell type-specific and induced by a wide variety of stimuli such as phorbol 12-myristate 13-acetate (PMA) and lipopolysaccharide [19-21]. The TNF-α protein is a multifunctional cytokine, and is involved in the regulation of a broad spectrum of biological processes [22-24]. The *TNF-α *gene appears to be silenced in HeLa cells, as evidenced by undetectable levels of mRNA by northern blot and protein by ELISA [25]. In the present study, we sought to engineer a HeLa cell line containing a targeted luciferase reporter in exon 1 of the *TNF-α *gene. We also sought to compare the patterns of *Renilla *luciferase (R-Luc) induction with endogenous *TNF-α *mRNA transcription between targeted and non-targeted cell lines in response to drug treatment. The production of a *TNF-α *gene-targeted reporter cell line will provide a sensitive and more predictive analytical tool for identifying molecules that modulate *TNF-α *gene transcription.

## Results and discussion

**Figure 1:**
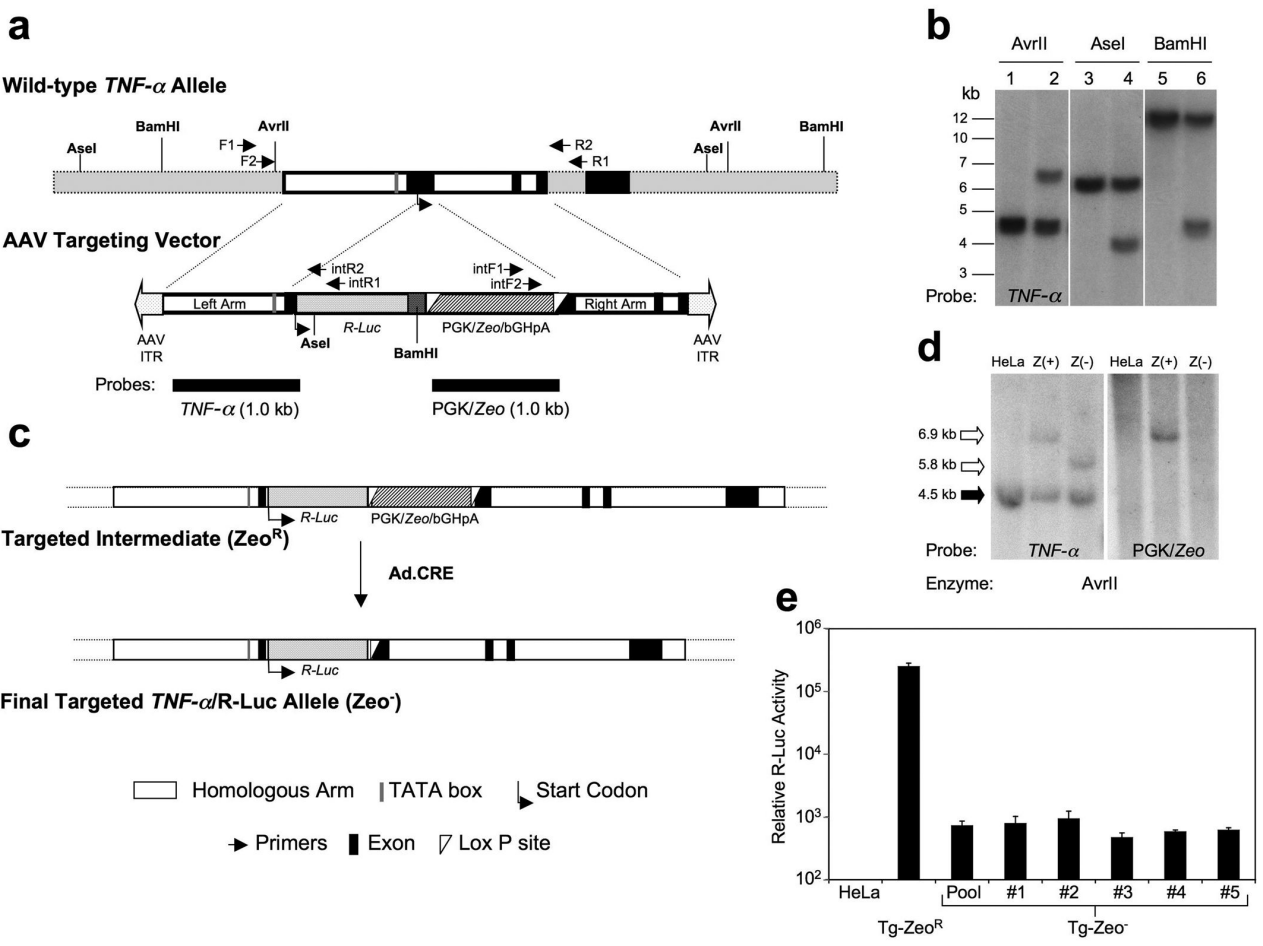
**Adeno-associated virus-mediated *TNF-α *gene targeting in HeLa cells**. (a) Genomic fragment containing the human *TNF-α *gene (top) and the recombinant adeno-associated virus (rAAV) *TNF-α *targeting vector (bottom). Arrows mark nested primers used for PCR screening of targeting events. Restriction sites and probes used for Southern blot confirmation of targeting events are also shown. The left and right homologous arms (Homologous Arm) in the AAV targeting vector contain endogenous sequence from the wild-type *TNF-α *allele. (b) Southern blot analysis of cells derived from the targeted intermediate clone Tg#28zeo^R^, using restriction sites and the *TNF-α *probe indicated in panel (a). Parental HeLa cell genomic DNA is shown in lanes 1, 3 and 5 and targeted clone Tg#28zeo^R ^genomic DNA in lanes 2, 4 and 6. (c) Strategy used to generate the final *TNF-α *targeted R-Luc reporter cell line by LoxP/Cre-mediated excision of the Zeocin selection cassette. (d) Southern blot analysis of the zeocin-resistant targeted intermediate clone (Tg#28zeo^R^, marked as Z^+^), and the Zeocin-sensitive targeted cell line (Tg#28zeo^-^, marked as Z^-^). Arrows to the left of the blots indicate the DNA fragments cut from non-targeted (solid) and gene-targeted (open) *TNF-α *alleles. (e) *Renilla *luciferase (R-Luc) reporter activity in the *TNF-α *targeted intermediate clone Tg#28zeo^R^, the Tg-Zeo^- ^cell pool following excision of the selection marker, and from 5 individual Tg-Zeo^- ^clonal cell lines (#1–5) isolated from the Tg-Zeo^- ^cell pool. Results represent the mean (+/-SEM, *N *= 4). One-way ANOVA demonstrated no significant difference (*p *> 0.05) between the targeted cell pool and the five individual targeted clonal cell lines isolated from the pool.

An rAAV targeting vector (AV.TNF-RL.targ) was generated to facilitate fusion of the *Renilla *luciferase (R-Luc) reporter gene to the *TNF-α *gene locus in HeLa cells (Figure 1a). The vector harbors a 2.1 kb genomic DNA fragment from the *TNF-α *locus, which was split into left and right homologous arms by the insertion of a R-Luc cDNA and loxP sites that flank a phosphoglycerate kinase (PGK) promoter-driven Zeocin expression cassette. The insertion site in exon 1 is immediately downstream of the *TNF-α *start codon, fusing the R-Luc gene in-frame to the *TNF-α *transcript. Since the left homologous arm of the targeting vector encodes the *TNF-α *core promoter and contains other regulatory elements necessary for initiation of transcription, we are able to compare reporter expression profiles between the targeted and non-targeted cell lines, the latter of which are derived from the random integration of AV.TNF-RL.targ in HeLa cells. A Zeocin-resistant gene serves as a selectable marker for clonal expansion of cells in which the rAAV genome has been stably integrated. Enrichment of stably integrated cells is required for this type of insertional gene targeting.

HeLa cells were infected with AV.TNF-RL.targ (Figure 1a) and re-plated for clonal expansion under Zeocin selection. Zeocin-resistant colonies were picked and transferred to replicate 96-well plates. Cells in replica plates were lysed for PCR screening with two sets of primers, which hybridize to sequences outside the right and left targeting arms and inside the exogenous insert. Clone #28 was identified as a positive targeted clone, from 192 clones screened, and its left-side PCR product was cloned into the pBlunt4PCR vector (Invitrogen) for sequence confirmation. Sequencing results revealed the presence of both the non-virus-derived flanking sequences and the expected in-frame fusion of the R-Luc cDNA in the *TNF-α *gene (data not shown). This positive clone (designated as Tg#28zeo^R^) was expanded and the genomic DNA was analyzed by digestion with several restriction enzymes (Figure 1b; lanes 2, 4 and 6). Genomic DNA from parental HeLa cells was used for comparison (Figure 1b; lanes 1, 3 and 5). The Southern blot was probed with *TNF-α *left arm-homologous sequences. The additional bands observed in the digested Tg#28zeo^R ^samples (Figure 1b; lanes 2, 4 and 6) are indicative of targeted insertion of the R-Luc cDNA at the *TNF-α *gene locus. No additional random vector integration was observed (Figure 1b).

The exogenous PGK promoter and transcription of the Zeocin gene could affect the transcriptional activity of the targeted *TNF-α *gene. To eliminate any possible artificial induction in R-Luc activity, the selection cassette was removed from the targeted intermediate, Tg#28zeo^R ^(Figure 1c). Flanked by a pair of LoxP sites, the PGK-Zeocin cassette can be easily excised from the targeting AAV genome. Cre recombinase-mediated excision was used to remove this selection cassette from the targeted Tg#28zeo^R ^line and also from the non-targeted cell lines that harbor random integrations of the targeting virus. A recombinant adenoviral vector, Ad.Cre, was used to deliver Cre recombinase to the cells. Southern blot analysis with probes for *TNF-α *and PGK/Zeo demonstrated that Ad.Cre infection resulted in loss of the selection cassette from the targeted intermediate, producing the final *TNF-α *reporter cell line, Tg#28zeo^- ^(Figure 1c and 1d).

Individual clones expanded from a single cell were isolated from the Zeocin-sensitive cell pool by limited dilution. Five independent lines were randomly selected and basal levels of R-Luc expression among these was compared. No apparent differences were observed between individual Tg#28zeo^- ^lines, and the expression levels were very similar to that in the original cell pool (Figure 1e). However, basal R-Luc activity in the targeted intermediate was more than 300-fold higher than in the clones lacking the Zeocin selection cassette (Figure 1e). Thus, as predicted, this selection marker enhanced transcription from the *TNF-α *gene locus, arguing that R-Luc activity in Tg#28zeo^- ^cells should more closely reflect endogenous *TNF-α *gene regulation than reporter activity in Tg#28zeo^R ^cells.

Current *TNF-α *reporter vectors contain only about 1.0 kb of core promoter located upstream of the *TNF-α *gene (Invitrogen Corp., Carlsbad, CA; Panomics, Inc., Fremont, CA). In addition, these plasmid-based *TNF-α*/reporter constructs are randomly inserted into the host cell genome following transfection. In theory, the fidelity of *TNF-α *gene expression in these randomly integrated reporter cell lines may be influenced by missing regulatory sequences (enhancer and/or silencer) not part of the 1.0 kb core promoter of the *TNF-α *gene [20,26,27]. Indeed, recent studies have demonstrated that the regulation of *TNF-α *expression involves distal enhancers located over a 12 kb region, and that these enhancers interact to form a novel double-loop chromatin configuration. This structure circularizes the *TNF-α *gene and thereby facilitates transcription [28-30]. Moreover, epigenetic regulation is important for the control of *TNF-α *transcription [31] and key epigenetic modifications at the *TNF-α *locus may be missing from the regulatory elements governing reporter expression in the randomly integrated reporter cell lines. In addition to the lack of intact endogenous regulatory elements and epigenetic modifications associated with the self-contained *TNF-α *core promoter/reporter gene, expression of the randomly integrated reporters is very likely to be influenced by both genetic and epigenetic features associated with the insertion site, in a very unpredictable manner. Thus, we hypothesized that the *TNF-α *gene would be an ideal platform to test whether targeted reporter expression more closely reflects endogenous gene expression patterns than randomly integrated reporters.

**Figure 2:**
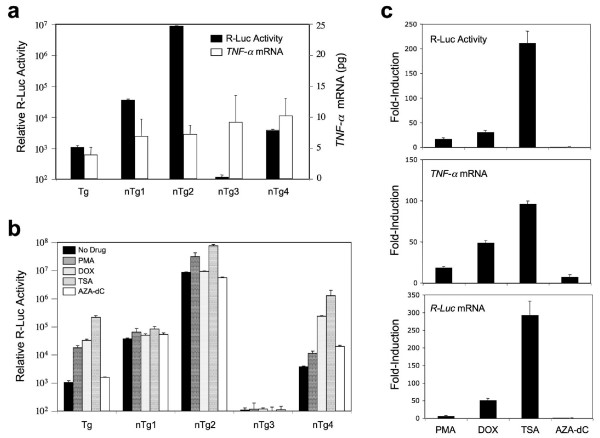
***Renilla *luciferase activity and *TNF-α *mRNA expression in *TNF-α *targeted and non-targeted reporter cell lines**. (a) Basal R-Luc reporter activity and *TNF-α *mRNA levels in the *TNF-α *targeted (Tg) cell line and in four non-targeted (nTg #1–4) cell lines. (b) Relative R-Luc activity in the targeted cell line (Tg) and in four non-targeted cell lines following treatment with the *TNF-α *transcriptional activator PMA (5 ng/ml), DOX (5 μM), TSA (100 ng/ml) or Aza-dC (5 mg/ml). (c) The drug-induction profiles of R-Luc activity (top), *TNF-α *mRNA expression (middle), and R-Luc mRNA expression (bottom) in the Tg cell line. Values represent the mean (+/-SEM, *N *= 4).

To test this hypothesis, we isolated 18 non-targeted *TNF-α*/R-Luc reporter clones in which the PGK-Zeocin cassette had been excised by Ad.Cre infection. We then compared basal R-Luc activity in the targeted and non-targeted reporter lines. Activity varied widely among the non-targeted lines relative to that in the targeted line. Four non-targeted (nTg) lines representing the range of basal R-Luc activity were selected for additional comparison to the Tg#28zeo^- ^targeted clone (Tg) (Figure 2a). *TNF-α *mRNA was purified from the reporter lines and quantified by TaqMan PCR (Figure 2a). Basal *TNF-α *mRNA levels were similar in all of the nTg lines, whereas the level in the Tg line was somewhat lower. Reduced expression in the Tg line was most likely due to the disruption of one *TNF-α *allele as a consequence of the in-frame insertion of the R-Luc cDNA. Tg and nTg cell lines were then treated with known inducers of *TNF-α *expression. Drugs representing different activation pathways were used: the protein kinase C activator phorbol 12-myristate 13-acetate (PMA) [32,33]; the DNA topoisomerase II inhibitor doxorubicin (DOX) [34,35]; the histone deacetylase inhibitor trichostatin A (TSA) [31,36]; and the DNA methylation inhibitor 5-aza-2'-deoxycitidine (Aza-dC) [31]. The extent of drug-induced reporter activity varied dramatically among the nTg cell lines tested – ranging from little (nTg2) or no induction (nTg1 and nTg3) to an induction profile similar to that in the Tg cell line (nTg4) (Figure 2b). However, even where such similarities existed, differences between the Tg and nTg4 lines were apparent. Drug-induced changes in *TNF-α *and R-Luc mRNA expression in the Tg line were then quantitated by TaqMan PCR and compared with R-Luc activity in the Tg line (Figure 2c). The induced patterns of both the *TNF-α *and R-Luc mRNAs closely reflected the patterns of R-Luc protein activity following drug treatment, indicating that reporter expression accurately reflects endogenous *TNF-α *gene expression in the Tg clone.

Throughout the approximately one-year study period, the basal level of R-Luc activity remained low and the levels of PMA- and TSA-induced reporter expression remained constant in the targeted cell line (data not shown). However, we did not conduct a systematic analysis of drug-induced reporter activity at regular intervals during this time. Nevertheless, while the long-term affects from integration of reporter gene sequences are unknown, our data do not indicate a progressive transcriptional silencing of the R-Luc gene.

**Figure 3:**
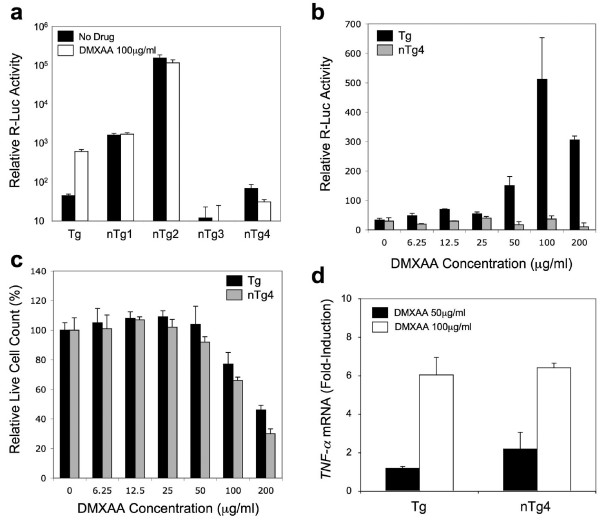
**DMXAA treatment of *TNF-α *targeted and non-targeted R-Luc reporter cell lines**. (a) Relative R-Luc activity in the targeted (Tg) and non-targeted (nTg #1–4) reporter cell lines in the absence or presence of DMXAA (100 μg/ml). (b) Dose-response profile for R-Luc activity following treatment of the Tg and nTg4 cell lines with DMXAA. (c) Dose-response profile for DMXAA-induced cytotoxicity in Tg and nTg4 cell lines. (d) Induction of *TNF-α *mRNA in the Tg and nTg4 cell lines following treatment with DMXAA. Despite the fact that DMXAA only induces R-Luc reporter activity in the Tg cell line, DMXAA induced *TNF-α *mRNA to equivalent levels in both cell lines. Values represent the mean (+/-SEM, *N *= 4).

We next probed potential regulatory differences between Tg and the nTg cell lines further by investigating their responsiveness to 5,6-Dimethylxanthenone-4-acetic acid (DMXAA) [37]. This is an agent that induces vascular permeability and tumor cell death in human solid tumors by activating *TNF-α *transcription [38,39] and is currently in Phase II clinical trials [40]. At a fixed drug concentration, DMXAA-induced R-Luc activity was observed in the Tg cell line but not in any of the tested nTg cell lines (Figure 3a). In DMXAA dose-response studies, R-Luc activity was induced in the Tg line by as much as ~10-fold, while induction in the nTg4 line (which was the nTg line that most closely mirrored Tg line responses by other drugs, Figure 2b) was insignificant (Figure 3b). This differential drug-based induction was not due to cell line-dependent differences in cellular toxicity (Figure 3c). Moreover, little to no difference was observed when comparing the up-regulation of *TNF-α *mRNA following DMXAA treatment in the Tg and nTg4 lines (Figure 3d). These data suggest that the 1.0 kb *TNF-α *core promoter region does not encode the DMXAA response element(s).

**Figure 4:**
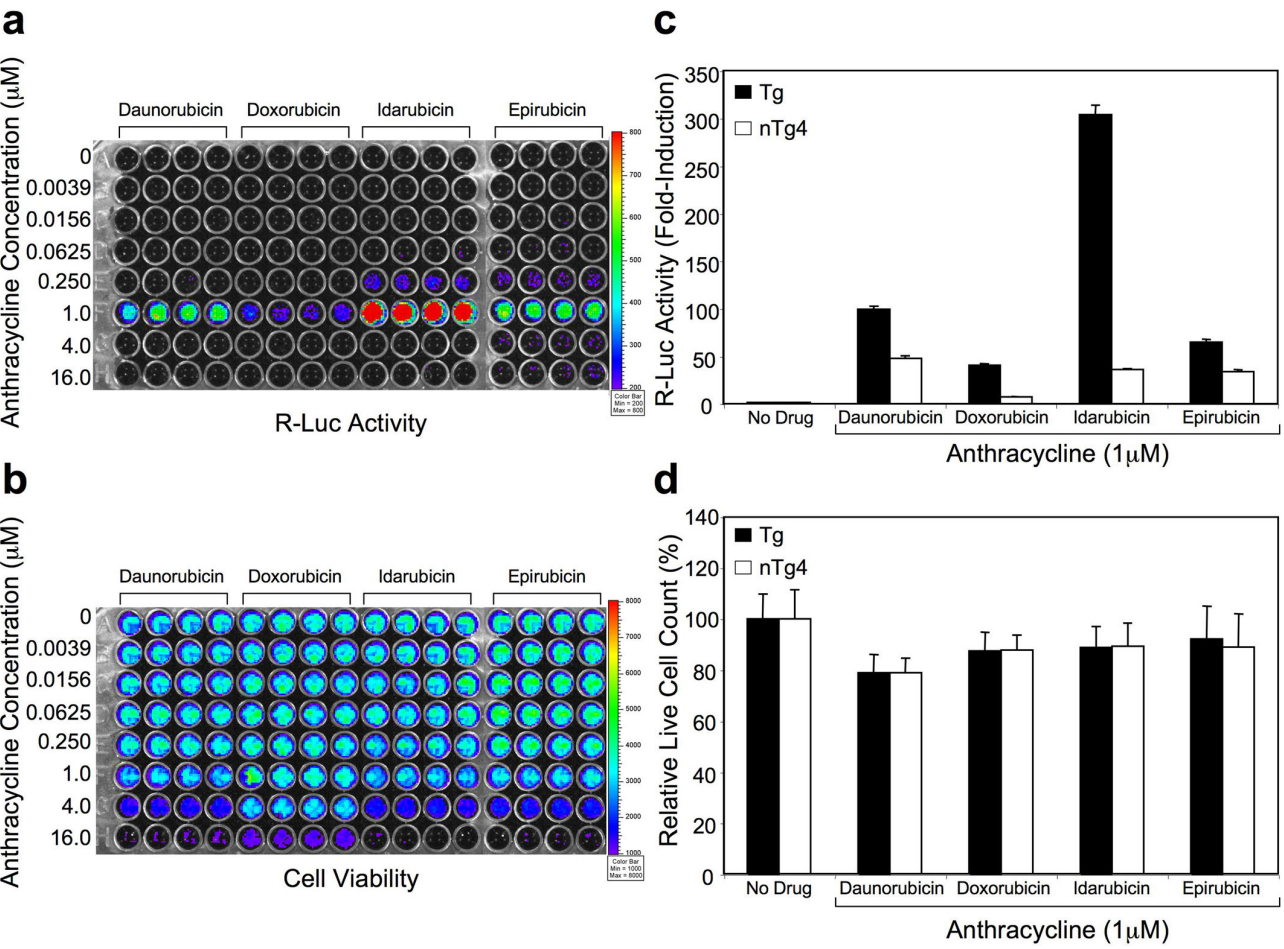
**Anthracycline treatment of *TNF-α *targeted and non-targeted R-Luc reporter cell lines**. (a) Dose-response profile of R-Luc activity in the targeted (Tg) cell line treated with four closely related anthracycline antibiotics. Cells treated with Epirubicin were grown on a separate plate. Luciferase activity was assessed using biophotonic imaging on the IVIS. (b) Dose-response profile of cell viability in the Tg line following anthracycline treatment. The same culture plates that are shown in 'a' are also shown in 'b'. Fluorescence was assessed using biophotonic imaging on the IVIS. (c) Relative R-Luc activity in targeted (Tg) and non-targeted (nTg4) cell lines following treatment with a fixed concentration of the different anthracyclines (1 μM). (d) Anthracycline-induced cytotoxicity in the Tg and nTg4 cell lines was similar. Values represent the mean (+/-SEM, *N *= 4).

Anthracycline antibiotics are also known activators of *TNF-α *promoter transcription. Dose-response studies with four closely related anthracycline antibiotics demonstrated a pronounced up-regulation of R-Luc activity in the Tg cell line at a drug concentration of 1 μM (Figure 4a). Anthracycline exposure did not appear to significantly reduce cell viability in the Tg line at this drug concentration (Figure 4b). Therefore, both Tg and nTg4 cell lines were treated with anthracyclines at 1 μM drug and assayed for R-Luc activity. Differential R-Luc activity was evident between these cell lines, most notably following Idarubicin treatment (Figure 4c). Indeed, Idarubicin induced R-Luc activity ~300-fold in the Tg line, but only ~50-fold in the nTg4 line – representing a 6-fold difference in induction between the Tg and the nTg4 cell lines. Differential induction between Tg and nTg4 cell lines was also observed following treatment with daunorubicin, doxorubicin and epirubicin. Again, these differences were not due to differences in anthracycline-induced cell death in the Tg and nTg4 cell lines (Figure 4d). Rather, we attribute the differences in R-Luc reporter activity to unique genetic and/or epigenetic features of the endogenous *TNF-α *gene locus. We also conclude that targeted reporter cell lines may be superior tools for screening drugs that modulate the transcriptional activity of target genes. The use of such cell lines with biophotonic imaging as presented here (Figure 4a and 4b) may be extremely useful for multi-parameter HTS to identify novel therapeutics.

## Conclusion

We conclude that gene-targeted reporter cell lines may more accurately index endogenous gene expression to facilitate predictive cell-based screening for drug discovery.

## Methods

### Cell culture, virus production and chemical

The human cervical adenocarcinoma cell line HeLa (ATCC CCL-2) was purchased from American Type Culture Collection (ATCC), and the cells were cultured in DMEM supplemented with 10% FBS. The Cre recombinase-expressing adenovirus vector, Ad.Cre, was obtained from the Vector Core of the Gene Therapy Center at The University of Iowa. PMA, DOX, Daunorubicin, Idarubicin, epirubicin, TSA and Aza-dC were purchased from Calbiochem. DMXAA was purchased from Sigma.

### Isolation of TNF-α genomic DNA and generation of the TNF-α targeting AAV proviral vector

A 2.8 kb *TNF-α *DNA fragment was amplified by PCR, using AccuPrime Pfx supermix (Invitrogen), from genomic DNA extracted from cultured HeLa cells. Cloning primers were designed based on the published human *TNF-α *sequence (Gene Bank ID: AB202113). The forward primer used was 5'-GAGCTGTGGGGAGAACAAAAGGA-3' and the reverse primer used was 5'-TTGGCCCTTGAAGAGGACCTG-3'. The *TNF-α *start codon is located in the center of the PCR product, and 1.32 kb of the promoter and 5'untranslated sequences were included. The PCR product was cloned into the pBlunt4PCR vector using a Topo cloning kit (Invitrogen) and its identity confirmed by DNA sequencing. The resultant plasmid was designated pTopo-TNF2.8. We constructed a PGK promoter-driven Zeocin expression cassette by replacing the neomycin-resistant gene in pPGKneo with the zeocin resistant gene, retrieved from pSV40/Zeo (Invitrogen). The resultant pPGKzeo plasmid was also flanked by a pair of LoxP sites. 1.2 kb *Renilla *luciferase cDNA, plus an SV40 polyadenylation signal, was retrieved from pRL-SV40 (Promega) and linked to the 5'end of the PGK promoter in the plasmid pPGKzeo to obtain the cloning intermediate pRL-PGKzeo. 1.0 kb of the left homologous arm containing the *TNF-α *promoter and the first translation start codon was amplified from the plasmid clone pTopo-TNF2.8 using the forward primer subLF (5'-cccaagcttAGAGCCTCCAGGACCTCCAGGTAT-3'; lower-case font indicates the flanking restriction enzyme sites for cloning) and the reverse primer subLR (5'-aactttcgaagt**CAT**GGTGTCCTTTCCAGGG-3'; bold font indicates the start codon). The PCR product was cut with *Hind*III and *BstB*1 and inserted into the plasmid pRL-PGKzeo, which resulted in the R-Luc cDNA being fused in frame to the *TNF-α *gene at the 3' end of the left homologous arm. The 1.0 kb right homologous arm was also amplified from the plasmid pTopo-TNF2.8, using the forward primer subRF (5'-cgagctaGCACTGAAAGCATGATCCGGG-3') and the reverse primer subRR (5'-ccatcgatGGGTTCGAGAAGATGATCCTGAAG-3'). The PCR product of the right arm, the DNA fragment containing the R-Luc-fused left arm and the PGK-Zeocin selectable marker were assembled and finally cloned into an AAV2 proviral plasmid, giving rise to a vector that harbors 2.0 kb of *TNF-α *genomic DNA fused in frame to the R-Luc cDNA and with a zeocin cassette inserted at the center. The AAV proviral vector was constructed in our laboratory with the AAV-2 inverted terminal repeats (ITRs) kindly provided by Targeted Genetics (Seattle, WA). The rAAV-2-targeting virus (AV.TNF-RL.targ, see Figure 1a) was produced as previously described, using a triple plasmid transfection procedure in 293 cells, and purified over an iodixanol cushion followed by ion exchange HPLC [41]. The genome size of this single-stranded rAAV targeting vector, including the ITRs, is 4.7 kb.

### Gene targeting and screening of homologous recombinants

5 × 10^5 ^HeLa cells were cultured in 60 mm dishes and infected with AV.TNF-RL.targ at a multiplicity of infection (MOI) of 100,000 particles per cell. On day 1 post-infection, HeLa cells were re-plated onto ten 100 mm dishes and selected in medium containing 150 μg/ml Zeocin for 16 days to allow for the expansion of Zeocin-resistant clones. One hundred and ninety-two well-separated colonies were picked, and expanded clonally in two 96-well plates. PCR screening was performed on one confluent replica plate using primer sequences outside the left vector homology arm and anchored within the R-Luc cDNA. Cells in the 96-well plates were then lysed by the addition of 10 μl per well of lysis buffer (50 mM KCL, 1.5 mM MgCl_2_, 10 mM Tris, pH 8.5, 0.5% NP40, 0.5% Tween 20), and 1/10 of the resultant cell lysate (1 μl) was used for PCR. The first round PCR primer set was as follows: F1 (5'-GGAAGCAAAGGAGAAGCTGAGAAGA-3') and intR1 (5'-AAGCGAAGGAGCAAAGCTGCTA-3'). One-fiftieth of each first-round PCR product was then used as a template for the second round of PCR with the following primers: F2 (5'-GCTCTGAGGAATGGGTTACAGGAG-3') and intR2 (5'-CCCAATCATGGCCGACAAAA-3'). The positive clones were confirmed by another nested PCR reaction targeting the right arm of the integration site, using the first round primer set: intF1 (5'-ACGTGACCCTGTTCATCAGCG-3') and R1 (5'-CGAGTCCTTCTCACATTGTCTCCAA-3'); and the second round nested primer set: intF2 (5'-TCGGAGGTCGTGTCCACGAACTT-3') and R2 (5'-CCTAGCCCTCCAAGTTCCAAGACA-3'). A single clone that was PCR-positive for both the left and right arms of the predicted integration event was expanded to 24-well plates, and eventually to 100 mm dishes, to generate sufficient cells for the preparation of genomic DNA and Southern blot confirmation of the integration event. Genomic DNAs were digested with either *Ase*I or *BamH*I (both of which are unique sites in the targeting vector) and *Avr*II (which does not cut within the targeting vector). Southern blotting was carried out with a P^32^-labeled *TNF-α *left arm probe or PGK/Zeo probe (see Figure 1). LoxP/Cre-mediated cassette excision was used to remove the PGK/Zeo cassette from the Zeocin-resistant targeted intermediate and the AAV integrated non-targeted cells. Zeocin-resistant cells were infected with adenovirus encoding Cre (Ad.Cre) at 500 MOI per cell. Half of the cells were cultured and expanded in normal culture medium, and the remaining cells were cultured in Zeocin-containing medium to confirm the loss of drug resistance associated with Cre-mediated excision. Clonal cell lines were derived from cells grown in absence of Zeocin by limiting dilution. Southern blot analysis with *TNF-α *and PGK/Zeo probes was carried out to confirm cassette removal.

### Renilla luciferase activity assay and drug-induction

Enzyme activity was determined using the *Renilla *Luciferase Assay System (Promega) in a 20/20 luminometer equipped with an automatic injector (Turner Biosystems). Parental, targeted, and non-targeted HeLa cells were treated with different chemicals for specific periods of time prior to assaying luciferase activity. The drug induction period for PMA, TSA, DMXAA, and the anthracycline antibiotics (daunorubicin, doxorubicin, idarubicin and epirubicin) was 24 hours, and the period for Aza-dC was three days. Cells were dispensed onto 6-well or 24-well plates one day before drug addition. Cells (2 × 10^5^) were lysed in 100 μl *Renilla *luciferase lysis buffer, and 1/10 of this cell lysate was assayed for luciferase activity. Four parallel samples were tested for each drug treatment. Cellular toxicity was assessed using the CellTiter-Blue Cell Viability Assay kit from Promega on the IVIS Biophotonic Imaging system, according to the manufacturer's instructions. In separate dose response experiments, *Renilla *Luciferase activity was also assessed using the IVIS Biophotonic Imaging system.

### RNA isolation and TaqMan-based PCR quantification

Total RNA was isolated from 1 × 10^6 ^cells, with or without drug treatment, and prepared using the RNAprotect Cell Reagent and the RNeasy Plus Mini kit (QiaGen). Total RNA samples eluted in a volume of 30 μl were used directly for reverse transcription (RT) or purified using the Micro-FastTract 2.0 mRNA Mini kit (Invitrogen) to enrich for polyA mRNA. The mRNA samples were resuspended in 10 μl RNase-free water. RT was performed using the High Capacity cDNA Reverse Transcription kit (ABI), through random priming. Either one-sixth (5 μl) of each total RNA sample or the entire volume of each mRNA sample (10 μl) was used for RT in a final reaction volume of 100 μl. The cDNA product (10 μl) from the RT reaction was used for TaqMan PCR quantification in a final reaction volume of 50 μl, using the TaqMan Universal PCR Master Mix (Applied Biosystems). A 20× mix of primers and FAM-labeled probe for the human *TNF-α *gene expression assay were purchased through ABI's Gene Expression Assay-on-Demand (Assay ID: Hs00174128_m1). The 20× mix of primers and FAM-labeled probe for the R-Luc expression assay were custom-ordered from ABI (Forward: 5'-GTGAAGTTCGTCGTCCAACATT-3'; Reverse: 5'-AACGTC AGGTTTACCACCTTTTACT-3'; TaqMan probe: 5'-FAM – CCTCGTGAAATCCCG). The housekeeping gene GAPDH was used for sample normalization, and the primer-limited VIC-labeled internal control for the GAPDH assay was also purchased from ABI. Relative quantification was performed using the TaqMan assay, and these data were collected and analyzed using a ABI-Prism 7900 HT Real-Time PCR Sequence Detection Systems (SDS v2.3). Conditions for the PCR reactions were 2 min at 50°C, 10 min at 95°C, and 40 cycles each consisting of 15 sec at 95°C and 1 min at 60°C. The experiments were performed in triplicate wells in a singleplex format when data were compared by the relative standard curve method, or in a multiplex format for both the FAM and VIC signals when data were analyzed by the comparative CT method.

## Authors' contributions

ZY contributed to the design of the study, the collection, analysis and interpretation of data and preparation of the manuscript. DL participated in the collection and analysis of data. JFE and GHL contributed to the conception and design of the study, analysis and interpretation of data and manuscript preparation. All authors read and approved the final manuscript.
